# Deep Feature Transfer Learning in Combination with Traditional Features Predicts Survival Among Patients with Lung Adenocarcinoma

**DOI:** 10.18383/j.tom.2016.00211

**Published:** 2016-12

**Authors:** Rahul Paul, Samuel H. Hawkins, Yoganand Balagurunathan, Matthew B. Schabath, Robert J. Gillies, Lawrence O. Hall, Dmitry B. Goldgof

**Affiliations:** 1 Department of Computer Science and Engineering, University of South Florida, Tampa, Florida;; 2 Department of Cancer Imaging and Metabolism, H. Lee Moffitt Cancer Center & Research Institute, Tampa, Florida; and; 3 Department of Cancer Epidemiology, H. Lee Moffitt Cancer Center & Research Institute, Tampa, Florida

**Keywords:** pre-trained CNN, transfer learning, deep features, computed tomography, symmetric uncertainty, lung cancer, adenocarcinoma, deep neural network

## Abstract

Lung cancer is the most common cause of cancer-related deaths in the USA. It can be detected and diagnosed using computed tomography images. For an automated classifier, identifying predictive features from medical images is a key concern. Deep feature extraction using pretrained convolutional neural networks (CNNs) has recently been successfully applied in some image domains. Here, we applied a pretrained CNN to extract deep features from 40 computed tomography images, with contrast, of non-small cell adenocarcinoma lung cancer, and combined deep features with traditional image features and trained classifiers to predict short- and long-term survivors. We experimented with several pretrained CNNs and several feature selection strategies. The best previously reported accuracy when using traditional quantitative features was 77.5% (area under the curve [AUC], 0.712), which was achieved by a decision tree classifier. The best reported accuracy from transfer learning and deep features was 77.5% (AUC, 0.713) using a decision tree classifier. When extracted deep neural network features were combined with traditional quantitative features, we obtained an accuracy of 90% (AUC, 0.935) with the 5 best post-rectified linear unit features extracted from a vgg-f pretrained CNN and the 5 best traditional features. The best results were achieved with the symmetric uncertainty feature ranking algorithm followed by a random forests classifier.

## Introduction

Lung cancer is the most common cause of cancer-related deaths in the USA (1). Early detection of cancer results in improved patient outcomes. Radiological imaging modalities, such as computed tomography (CT) and magnetic resonance imaging, can help in early detection, diagnosis, and management of cancer. Radiomics (33) is a process of extracting quantitative features for computer-aided image analysis on high-quality medical images (eg, magnetic resonance imaging, CT). The process involves extraction of various quantitative features from a region of interest (2) (intensity, shape, or texture) and use of those (and potentially other) features to provide actionable information.

Traditional quantitative features (30, 31, 32, 36, 37) may be insufficient for tumor classification; therefore, features extracted from a deep neural network may prove helpful. Recently, deep feature extraction from a convolutional neural network (CNN) has shown good classification performance in the machine learning field. Artificial neural networks (ANN), which are inspired by biological neural networks, have been used for classification and prediction. ANNs consist of several layers—input, hidden, and output layers—and there can be multiple hidden layers. Hidden and output nodes contain an activation function. There are some issues when using ANNs. Every consecutive layer is interconnected; therefore, the number of weights will rapidly increase with more layers, which, in turn, will affect the learning rate. For images, this problem can be dealt with by using several small filters on the input and subsampling the space of filter activations until there are sufficient high-level features. Neural networks using filters are called CNNs, which are currently a highly effective approach for image classification and recognition tasks.

The predecessor of convolutional networks the “neocognitron” was proposed by Fukushima (3) in 1980. In a major advancement, backpropagation allowed visual recognition by deep convolutional networks (LeCun et al.) (4). This was further simplified and advanced by Simard et al. (5) for larger data sets. Krizhevsky et al. (6) achieved improved performance by using a CNN for large-scale image classification when using an image database with millions of examples, ImageNet. Girshick et al. (7) used supervised pretraining, when data was scarce, with fine-tuning in a specific domain. Donahue et al. (8) examined whether the features extracted using a pretrained CNN could be used for various classification tasks. Pretraining reduced the overfitting problem and made the optimization easier (9). The use of pretrained CNNs is based on transfer learning (35), which means using previously learned knowledge to improve the accuracy for a new task.

Based on transfer learning methodologies, in our current study, we used 3 existing CNN models pretrained on ImageNet (10) to extract deep features from lung tumors. Medical images are different from the ImageNet images of objects in natural scenes; therefore, in this study, we also showed that we could classify and predict outcomes from medical images using an ImageNet-trained CNN network. We compared our results with the results of Hawkins et al. (2), which were obtained based on traditional quantitative features. In this paper, we also experimented by mixing deep features with quantitative features. This work is an extension of Paul et al.'s study (11) with more experiments, analysis using both pre- and post-rectified linear unit (ReLU) features, and the use of multiple slices and more emphasis on mixed feature sets. Here, we obtained 90% accuracy (0.935 area under the curve [AUC]), which is a significant AUC improvement over the best previous results of 77.5% (AUC, 0.713) (11).

## Transfer Learning

Transfer learning (12,13) is an approach, by which a system can apply knowledge learned from previous tasks to a new task domain that is, in some way, related to the previous domain. This theory is inspired from the idea that people can intuitively use their previously learned experience to define and solve new problems.

In our study, we used a CNN pretrained on ImageNet. ImageNet (10), currently the largest data set for image classification and visual recognition, is an image database with >14 million images of >1000 object categories, organized according to the WordNet hierarchy. We have a relatively small data set (40 cases), which is very small to train a CNN that can have millions of weights to learn. Therefore, we used several different CNN architectures, which were previously trained on a subset of the current ImageNet database—MatConvNet-vgg-f, MatConvNet-vgg-m, and MatConvNet-vgg-s. There is a wide variety in the 1000 classes of objects in ImageNet, and we hypothesize that some useful texture features may exist.

When using the CNN as a feature extractor, we removed the last fully connected layer, which is the output layer. Feature values can be extracted as raw values or after they have been transformed by an ReLU, where an output x is mapped to max(0,x). The activation values that we got from the last hidden layer were the deep features (preReLU features, 4096). We also extracted the postReLU features (4096 feature vectors). Then, we can use these “deep features” for training and classification.

### Convolutional Neural Networks

A CNN (34, 38, 39, 40, 41, 42, 43, 44) is a type of multilayer feed-forward biologically influenced neural network. A CNN has several layers, which can be classified into the following 3 types: convolutional (compute the output of the connected local input region neurons), max pooling (subsampling the inputs), and fully connected layers (used in computing each class's activation). The input to a CNN is an n × n × m image, where n is the height and width and m is the number of channels, and there will be k convolutional filters of size a × a in the convolutional layer, where a < n. We have used the vgg-F, vgg-M, and vgg-S architectures of pretrained CNNs described in Chatfield et al.'s work (14). They have 5 convolutional layers followed by 3 fully connected layers. The details of these architectures are described in Tables 1 to 3. All the networks used Dropout in training, where some random weights were not allowed to change for an iteration. They also used ReLUs for the fully connected layers. More description about the CNN architecture can be found in Srivastava et al.'s (15) and Dumoulin et al.'s (16) studies. The pretrained CNN that we used in our study is implemented in a MATLAB toolbox named MatConvNet (17). The toolbox required the input image size to be a 224 × 224 RGB (red, green, and blue) image. Because, chest CT images do not have a color component, we modified the code to allow the grayscale images to be fed to the CNN. The voxel intensities of the CT images were first converted to the range [0, 255]. For the pretrained networks, the input images need to be normalized by average image first; in our experiment, we took only the information from the R channel, which has the lowest frequency, for normalization, and ignored the B and G channels. The deep features have been taken from the last layer before the outputs, the fully connected seventh layer, and we consider the activation values (both pre- and postReLU) that we are obtaining from this last hidden layer as deep features (dimensional feature vectors, 4096) for the input images.

**Table 1.** vgg-F Architecture

Conv 1	64 × 11 × 11 st. 4, pad 0
Conv 2	256 × 5 × 5 st. 1, pad 2
Conv 3	256 × 3 × 3 st. 1, pad 1
Conv 4	256 × 3 × 3 st. 1, pad 1
Conv 5	256 × 3 × 3 st. 1, pad 1
Full 6	4096 dropout
Full 7	4096 dropout
Full 8	1000 softmax

**Table 2.** vgg-M Architecture

Conv 1	96 × 7 × 7 st. 2, pad 0
Conv 2	256 × 5 × 5 st. 2, pad 1
Conv 3	512 × 3 × 3 st. 1, pad 1
Conv 4	512 × 3 × 3 st. 1, pad 1
Conv 5	512 × 3 × 3 st. 1, pad 1
Full 6	4096 dropout
Full 7	4096 dropout
Full 8	1000 softmax

**Table 3.** vgg-S Architecture

Conv 1	96 × 7 × 7 st. 2, pad 0
Conv 2	256 × 5 × 5 st. 1, pad 1
Conv 3	512 × 3 × 3 st. 1, pad 1
Conv 4	512 × 3 × 3 st. 1, pad 1
Conv 5	512 × 3 × 3 st. 1, pad 1
Full 6	4096 dropout
Full 7	4096 dropout
Full 8	1000 softmax

## Classifiers and Feature Selectors

We experimented with the 4 classifiers described below, as implemented in Weka (version 3.6.8) (18).

### Naïve Bayes

The naïve Bayes classifier (19), which is based on the Bayes' theorem, is a simple probabilistic classifier that does not have a complicated iterative parameter estimation. It makes an assumption that the input features are independent of each other, given the class variable. For estimating the classification result, it requires only a small amount of data in training and also performs well in case of categorical variables, which we do not currently use.

### Nearest Neighbors

A nearest neighbor classifier (20) is a lazy, nonparametric instance-based learning algorithm. The main idea behind this algorithm is to find how close a new example and stored examples are and to predict labels from the nearest examples. The closeness is measured using a distance function (eg, Euclidean distance, Manhattan distance, Minkowski distance, etc.), and the classification of a new example is based on a vote of the k nearest neighbors. In this approach, training is very fast and it does not lose information, but more comparisons and larger memory are required, if there is a large training set.

### Decision Trees

Decision trees (21) are a top-down, nonparametric classification algorithm. They are made up of the following 3 types of nodes: root node (no incoming edges), internal node or test node, and leaf node (decision nodes). The test at the root node splits the data set into smaller subsets of internal (or leaf) nodes based on some test conditions, and examples at internal nodes will continue being split by tests until a leaf node is created consisting of either pure or nearly pure examples. Leaf nodes are the decision nodes and contain the class labels. A small to medium-size decision tree is easy to comprehend and does not require any domain knowledge.

### Random Forests

Random forests (22) is a classification method that trains ensembles of decision trees and chooses the class by using majority voting among the trained trees. It uses bagging (creation of a new training data set by selection with replacement) and random selection of a test from the highest-ranked tests at an internal node of a tree. We used 100 trees and randomly chose from log_2_ (4096) + 1 = 13 features. It works quite effectively on large data sets.

With each classifier, we tried the following 2 filter feature selectors. This gave us 8 feature selector classifiers pairs.

### Relief-f Feature Selector

Relief-f (23–25) is a simple, noise-tolerant, effective feature selection algorithm for finding features with strong class dependencies. This approach uses a nearest neighbor algorithm on both the same class and the opposite class instances for ranking the features. It assigns more weight to the features that help differentiate between distinct classes. We used the top 5 and 10 features found by the algorithm for classification.

### Symmetric Uncertainty Feature Selector

Symmetric uncertainty (26) is a feature selection approach that can be used to rank the features by calculating the fitness between the features and the classes. It can be calculated using the ratio between the information gain of 2 features and the summation of entropy of those 2 features. We used the top 5 and 10 features found by the algorithm for classification.

## Experiments

### Data Set

**Figure 1.**
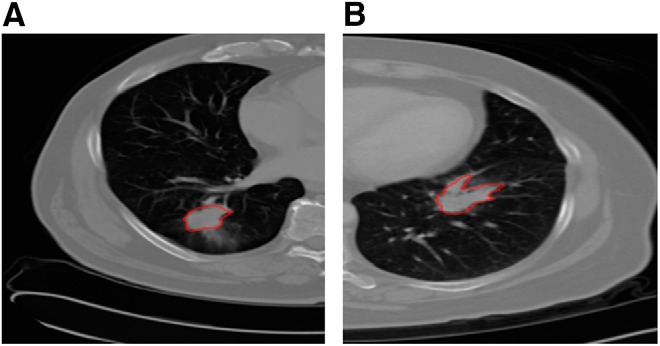
Example computed tomography (CT) slices of long-term (A) and short-term (B) survival groups with tumors outlined.

The data set used consisted of chest CT scans and associated clinical data in an institutional review board-approved study from the H. Lee Moffitt Cancer Center & Research Institute, Tampa, Florida. Eighty-one patients with >2 years of follow-up imaged with standard-of-care contrast-enhanced CT scans who had non-small cell cancer with biopsy-verified adenocarcinoma were used for survival time analysis. Most (24) scans of the subset of 40 used here were 5 mm thick slices and the second most (7) were at 6 mm. The others were scattered from 2 to 4 mm, with most at 3 and 4 mm. The scanners used were from GE and Siemens, with 1 exception. There was no observed relationship of classes with slice thickness or scanner. The data set is challengingly diverse. Patient data were de-identified and were distributed into 4 stages as follows: 32 cases in stage 1, 20 in stage 2, 25 in stage 3, and 4 cases in stage 4. The median survival time from diagnosis was 925 days. The 81 patients were divided into upper and lower quartiles based on the survival time. The lower quartile (class = −1) consisted of 20 patients surviving from 103 to 498 days, with a median survival time of 289 days. The upper quartile (class = 1) consisted of 20 patients surviving from 1351 to 2163 days, with a median survival time of 1551 days. We classified patients in the upper and lower quartiles as long- and short-term survivors, respectively. As such, we used the same 40 lung cancer cases as those analyzed by Hawkins et al. (2). Figure 1A presents an example of a patient from the long-term class (Left), and Figure 1B presents an example of a patient from the short-term class (Right). Lung region segmentation was performed using the Lung Tumor Analysis software suite from Definiens (27). Tumor identification was conducted by radiologists from H. Lee Moffitt Cancer Center & Research Institute. Finally, a region-growing algorithm by Gu et al. (28) was applied to segment the tumor region. Note that this is the same data set that was used by Hawkins et al. (2).

Table 4 shows the demographics of the data set and *P* values for differences in the classes where appropriate. There were no significant differences in demographics found between the examples in the 2 classes.

**Table 4.** Demographic Summary of Patients in the Data Set

Characteristics	Short Survival Class	Long Survival Class	*P* Value
Age, mean (SD)	69 (8.07)	64.45 (9.75)	0.1161 (Unpaired student *t*-test)
Sex, N (%)			
Male	12 (60%)	7 (35%)	0.2049 (Fisher exact test)
Female	8 (40%)	13 (65%)	
Race			
White	20 (100%)	20 (100%)	1 (Fisher exact test)
Black, Asian, and Others	0 (0%)	0 (0%)	
Ethnicity, N (%)			
Hispanic or Latino	1 (5%)	0 (0%)	1 (Fisher exact test)
Neither Hispanic/Latino and unknown	19 (95%)	20 (100%)	
Histology, N (%)			
Adenocarcinoma	20 (100%)	20 (100%)	
Squamous cell carcinoma	0 (0%)	0 (0%)	
Other, NOS, unknown	0 (0%)	0 (0%)	
Stage, N (%)			
I	4 (20%)	10 (50%)	
II	5 (25%)	5 (25%)	
III	10 (50%)	3 (15%)	
IV	1 (5%)	2 (10%)	
Carcinoid, unknown	0 (0%)	0 (0%)	
Tobacco Use, N%			
Moderate (1–2 PPD)	4 (20%)	4 (20%)	
Light (<1 PPD)	0 (0%)	1 (5%)	
HIST	12 (60%)	12 (60%)	
None	0 (0%)	3 (15%)	
Cigarettes Nos	4 (20%)	0 (0%)	

In our study, for each case of lower- and upper-quartile data, we chose the tumor slice that had the largest tumor area. This was to maximize the tumor in one slice given to the CNN for feature selection. One risk is if important characteristics are missing, such as the ground glass part of a tumor (in our data set, we did not have ground-glass tumors). We mitigated the risk by using multiple slices. We explored extracting deep features using a cropped image of the same size for each tumor or by using a rectangle that most closely fit the tumor. In both cases, the image must be resized to 224 × 224 for the CNN. We call the approach of directly warping a variable-size rectangle that is tightly fit to the tumor: “warping”.

In our study, we used the symmetric uncertainty and relief-f feature selectors on the training set while performing a leave-one-out cross validation. We calculated both accuracy (accuracy is the 0.5 probability threshold) and AUC (obtained by adjusting the probability threshold for each classifier). We tested the classification process with the following 4 classifiers: naïve Bayes, nearest neighbor, decision tree, and random forests. So, there were 8 feature selector/classifier pairs. For space, we report only a subset of best results (full results can be found in the Supplemental Appendix).

### Deep Feature Extraction from “Warped” Tumor Patches

**Figure 2.**
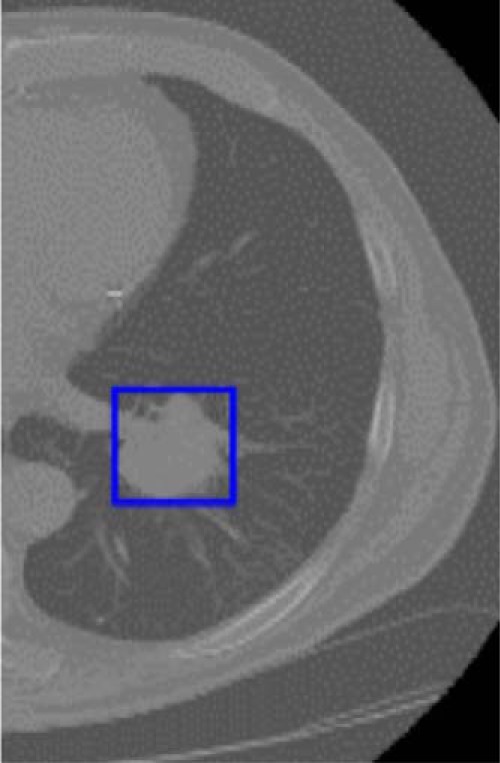
Example of lung patch used for the warped approach.

We created the tumor patch from the CT scan image by drawing a rectangular box that completely covered the tumor and we called it a “warped” tumor, as each starts with a different size and ends up transformed to the same size. This was performed on the slice in the axial plane with the largest tumor area. The sizes of the warped tumors were different for each case. Some interpolation was necessary to use a CNN with different-sized tumors; it may have unpredictable effects. It is possible the CNN compensates for the interpolation well, as it seems to do for camera images of different sizes. Because the pretrained CNN-F architecture requires a 224 × 224 input image size, we used bicubic interpolation to resize the tumor patches and then extracted deep features from them. Figure 2 shows an outlined tumor in a slice to be warped. The size of each deep feature vector from each tumor patch was 4096. As we obtained a large number of features from each tumor patch, which may not all be useful for classification, we used a feature selector for ranking the deep features and then classified the instances. The best result of 75% was obtained using preReLU features from a vgg-f CNN architecture and a random forests classifier in a leave-one-out cross validation, with 5 features using the symmetric uncertainty feature ranking algorithm on each fold.

Using postReLU features, the best result of 70% was obtained from a vgg-s CNN architecture and a random forest classifier in a leave-one-out cross validation with 10 features and the relief-f ranking algorithm on each fold. The classification was performed on both 5 and 10 features.

### Deep Feature Extraction from “Cropped” Tumor Patches

The size of each tumor patch is different; therefore, we explored the usage of different-sized windows on all tumors. We calculated the average height and width of the window that exactly encompassed the tumor. The average height and width is ∼40 pixels in the native resolution of the scan; as such, we chose a size of 40 × 40 window for automatically cropping the tumor patch from the center pixel of the tumor using the tumor mask.

**Figure 3.**
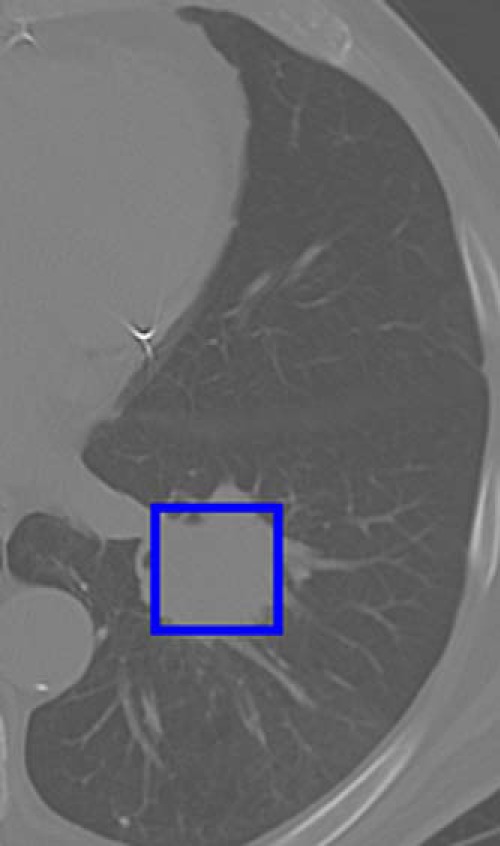
Example of lung patch used for the cropped approach.

In addition to an average-size window, a bigger window size of 56 × 56, which completely covered almost all tumors (only 6 tumor patches are bigger than 56 × 56), was used. This also provides some information on whether the area outside the tumor provides useful information for prediction. Figure 3 presents a cropped box for the same tumor as shown in Figure 2. The feature extraction and classification procedure is the same as the warped method. With the 40 × 40 cropped window method, we found a best accuracy of 75% using preReLU features from the vgg-f CNN architecture with a decision tree classifier in a leave-one-out cross validation with 5 features using the relief-f feature ranking.

Using the 40 × 40 cropped window method and postReLU features from the vgg-m CNN architecture, we found a best result of 82.5% with a decision tree classifier in a leave-one-out cross validation with 5 features using the symmetric uncertainty feature ranking. The classification was performed on 5 and 10 features, selected per fold.

With a 56 × 56 cropped window, we got a best result of 77.5% using preReLU features from the vgg-f CNN architecture with a decision tree classifier in a leave-one-out cross validation with 10 features using the symmetric uncertainty feature ranking.

With a 56 × 56 cropped window using postReLU features, we got a best result of 65% from the vgg-m CNN architecture with a decision tree classifier in a leave-one-out cross validation with 5 features using the symmetric uncertainty feature ranking. The classification was performed on both 5 and 10 features, which were selected per fold.

### Deep Feature Extraction from “Warped” Multiple Tumor slices

We used multiple slices for each patient to generate additional features for classification. For each patient, we selected the slice that had the largest tumor area and selected the slices just before and after the largest slice. We created the tumor patch by drawing a rectangular box that completely covered the tumor. The resizing of each small patch and the feature extraction and classification were done as previously described. The predictions of each slice were voted.

The best result using preReLU features was 85% with the vgg-f CNN architecture and a random forest classifier in a leave-one-out cross validation with 10 features using the symmetric uncertainty feature ranking algorithm on each fold.

An improved result of 87.5% was obtained using postReLU features from vgg-f CNN architecture and a random forest classifier in a leave-one-out cross validation with 5 features using the symmetric uncertainty feature ranking algorithm on each fold.

The classification was performed on both 5 and 10 features, selected per fold.

### Merging Deep Features with Quantitative Features

We merged the top 5 deep features from our method with traditional quantitative features from Hawkins et al.'s study (2). This is a new approach where we try to incorporate both deep features and quantitative features. We selected the top 5 features from each set using both symmetric uncertainty and the relief-f feature selector. There are 5 features selected per fold from both deep features and traditional quantitative features for a total of 10 features.

The best accuracy obtained using a single-slice approach (by merging deep and traditional quantitative features extracted from single-tumor slice) was 82.5% using the preReLU deep features from the vgg-f CNN architecture using a cropped window (56 × 56) and traditional quantitative features from Hawkins et al.'s study (2), a nearest neighbor classifier in a leave-one-out cross validation with 10 features, and the symmetric uncertainty feature ranking. From the cropped (40 × 40) single-tumor slice, using preReLU deep features from vgg-f CNN architecture and traditional quantitative features from Hawkins et al.'s study (2) with a naïve Bayes classifier in a leave-one-out cross validation with 10 features and the relief-f feature ranking, we obtained an accuracy of 82.5%.

The best accuracy obtained from the multiple-slice approach was 82.5% using the preReLU deep features from vgg-f CNN architecture with random forest classifier in a leave-one-out cross validation with 10 features and the symmetric uncertainty feature ranking.

The best accuracy obtained from the single-slice approach was 90% using the postReLU deep features from warped vgg-f CNN architecture and those mentioned in Hawkins et al.'s study (2) with naïve Bayes classifier in a leave-one-out cross validation with 10 features and relief-f feature ranking.

The best accuracy obtained from a multiple-slice approach was also 90% using the postReLU deep features from a vgg-f CNN architecture with a random forests classifier in a leave-one-out cross validation with 10 features and the symmetric uncertainty feature ranking.

Table 5 summarizes the best results that were obtained with deep features alone and with mixed features. Results are shown for single and multiple slices. The AUCs are included with the highest value of 0.935.

**Table 5.** Selected Results

**Pre-Trained CNN Architecture**	vgg-m (postReLU 5 Features)	vgg-m (postReLU 5 Features)	vgg-f (postReLU 5 Features)	vgg-f (postReLU Features)	vgg-f (postReLU Features)
**Feature Type**	Deep features	Deep features	Deep features	Mixed (Deep + Traditional quantitative) features	Mixed (Deep + Traditional quantitative) features
**Number of Slices**	Single	Single	Multiple	Single	Multiple
**Classifier Used**	Decision tree	Random forest	Random forest	Naïve bayes	Random forest
**Feature Selector Used**	Symmetric uncertainty	Symmetric uncertainty	Symmetric uncertainty	Relief-f	Symmetric uncertainty
**Number of Features**	5	5	5	10 (5 Deep + 5 Traditional quantitative image features)	10 (5 Deep + 5 Traditional quantitative image features)
**Accuracy**	82.5%	72.5%	87.5%	90%	90%
**AUC**	0.778	0.804	0.875	0.935	0.935

Abbreviations: CNN, convolutional neural network; ReLU, rectified linear unit; AUC, area under the curve.

We compared our best result with the result obtained from Hawkins et al.'s study (2). The previous method is based on the size, shape, and texture of the tumor patches obtained from the CT scan images. The best accuracy, solely using 219 traditional quantitative features, was 77.5% with an AUC of 0.713 using a decision tree classifier in a leave-one-out cross validation with 5 features using the relief-f feature ranking.

The best accuracy of our combined method was an improved 90% with an AUC of 0.935. The AUC difference between the traditional features and the combined approach is statistically significant at the *P* = .05 level.

## Discussion

The meaning of the deep features and potential correlation remains to be investigated. With the small amount of data, we could not show any statistical difference between using deep features, multiple slices, and the mixed-feature model with random forests other than the AUC of our traditional feature approach and the mixed-feature approach. Although the mixed and deep-feature approach showed >12% increase in accuracy, it is not a statistically significant increase with this small data set.

The stability of the deep features for the vgg-f CNN postReLU experiment where the best 5 features were used, as identified by the symmetric uncertainty feature selector, was investigated. The top feature was the same for all 40 trials. The second best feature was the same for 37 trials, and in the top 40 (at a different rank), it appeared 3 more times. Three more features appeared at least 26 times. So, the deep features had some stability.

A recent study (29) using the Lung Image Database Consortium data set showed that a classifier could predict whether a lung nodule was cancerous with an overall accuracy of 75.01% using different types of deep features than those used in this study. They used a 5-layered denoising autoencoder-trained network to extract features; 200 features extracted from layer 4 were given to a decision tree. Only deep features were used, which shows their potential.

## Conclusions

Recent advancements in CNNs have opened another path to extract features and analyze tumor patches from CT. Adding features of lung tumors from a CNN provides some potentially new features not in a nonexhaustive set of the usual quantitative features (eg, Haarlick, Laws, Wavelets). The tumors here are of different sizes and must be preprocessed before they are given to a CNN. In this paper, we used the transfer learning concept, in which previously learned knowledge is used in a new task domain. Here, we used CNNs pretrained on ImageNet to select features, which is faster than training a CNN (for which we need much larger data). In this study, we also showed that images from ImageNet, which are camera images of nonmedical objects and hence considerably different from lung cancer images, could be used for extracting useful features from the tumor patches. We used 3 different pretrained CNN architectures and extracted pre- and postReLU features. By using the pretrained CNN (vgg-f architecture) and preReLU features with a single slice, we obtained an accuracy of 77.5% using 10 features in predicting patients to be either short- or long-term survivors. In the multiple-slice approach, the best result of 85% using 10 features was obtained using preReLU features from the vgg-f CNN architecture. We experimented by merging the top 5 features from both a pretrained CNN (preReLU) and the traditional quantitative features approach and found that the best accuracy was 82.5% from a vgg-f architecture and using a nearest neighbor classifier in a leave-one-out cross validation with symmetric uncertainty feature ranking.

By using the postReLU features from a single slice using pretrained CNN (vgg-m architecture), we found an accuracy of 82.5% using 5 features. In the multiple-slice approach, the best result of 87.5% was obtained using postReLU features from vgg-f CNN architecture.

When we experimented by merging the top 5 features from both a pretrained CNN (postReLU) and the traditional quantitative features approach, using a single-slice approach, we found that the best accuracy was 90% from a vgg-f architecture using a naïve Bayes classifier in a leave-one-out cross validation with the relief-f feature ranking. Using the multiple-slice approach, we found that the best accuracy was 90% from vgg-f architecture using a random forest classifier in a leave-one-out cross validation with the symmetric uncertainty feature ranking.

The 90% accuracy (AUC, 0.935) is a big improvement over the previous best results of 77.5% (AUC, 0.713) with both “handcrafted” (traditional quantitative) features (2) and deep features (11) and 82.5% by mixing “handcrafted” and deep features (11). The AUC is statistically significantly higher than the previous best results. Our future work consists of tuning CNNs.

### Supplemental Materials

Supplemental Appendix:
