# Supplementary material for: Deep Feature Transfer Learning in Combination with Traditional Features Predicts Survival Among Patients with Lung Adenocarcinoma
Source: Tomography. 2016 Dec;2(4):388–95. doi: 10.18383/j.tom.2016.00211 (PMC5218828; doi:10.18383/j.tom.2016.00211)
Supplement: Supplemental Appendix: [file tom-00211-16-s001.pdf]

Deep Feature Transfer Learning in Combination with Traditional Features Predicts Survival among Patients with Lung Adenocarcinoma

Rahul Paul[1], Samuel H. Hawkins[1], Y. Balagurunathan[2], Matthew B. Schabath[2,3], Robert J. Gillies[2], Lawrence O. Hall[1], Dmitry B. Goldgof[1]

[1]Department of Computer Science and Engineering, University of South Florida, Tampa, Florida, USA
[2]Department of Cancer Imaging and Metabolism, H. Lee Moffitt Cancer Center and Research Institute, Tampa, Florida, USA
[3]Department of Cancer Epidemiology, H. Lee Moffitt Cancer Center and Research Institute, Tampa, Florida, USA

Abstract

Lung cancer is the most common cause of cancer related deaths in the US (1). It can be detected and diagnosed with the help of computed tomography (CT) images. For an automated classifier, identifying predictive features from medical images is a key concern. Deep feature extraction using pre-trained convolutional neural networks has recently been successful when applied in some image domains. In this paper, we applied a pre-trained convolutional neural network (CNN) to extract deep features from 40 contrast CT images of non-small cell adenocarcinoma lung cancer, combined deep features with traditional image features and trained classifiers to predict short and long term survivors. We experimented with several pre-trained CNNs and several feature selection strategies.

The best previously reported accuracy while using traditional quantitative features was 77.5% (16) (AUC 0.712) and was achieved by a decision tree classifier. The best reported accuracy from transfer learning and deep features was 77.5% (40) (AUC 0.713) as well and was achieved by a decision tree classifier. When we combined extracted deep neural network features along with traditional quantitative features we obtained an accuracy of 90% (AUC 0.935) with the five best post-relu features extracted from a vgg-f pre-trained CNN and the 5 best traditional features. The best results were achieved with the symmetric uncertainty feature ranking algorithm followed by random forest classifier.

Results: Here we present all the results obtained using various pre-trained CNN architectures and classifiers and feature selectors.

A. Results obtained from pre-relu features

TABLE IV. Accuracies from warped tumor patches using vgg-f network

Classifier used	***Symmetric uncertainty feature selector***	***AUC***	***Relief-f feature selector***	***AUC***
Naïve bayes (5 features)	72.5%	0.773	57.5%	0.566
Naïve bayes (10 features)	57.5%	0.575	60%	0.589
Nearest neighbor(5 features)	67.5%	0.675	70%	0.596
Nearest neighbor(10 features)	57.5%	0.566	62.5%	0.525
Decision tree(5 features)	67.5%	0.675	70%	0.724
Decision tree(10 features)	65%	0.654	62.5%	0.666
Random Forests(5 features)	**75%**	0.75	70%	0.77
Random Forests(10 features	72.5%	0.645	65%	0.654

Table V. Accuracies from cropped (40x40) tumor patches using vgg-f network

Classifier used	***Symmetric uncertainty feature selector***	***AUC***	***Relief-f feature selector***	***AUC***
Naïve bayes (5 features)	65%	0.617	55%	0.638
Naïve bayes (10 features)	47.5%	0.436	57.5%	0.575
Nearest neighbor(5 features)	45%	0.403	52.5%	0.513
Nearest neighbor(10 features)	55%	0.563	42.5%	0.253
Decision tree(5 features)	65%	0.654	**75%**	**0.75**
Decision tree(10 features)	60%	0.589	65%	0.617
Random Forests(5 features)	50%	0.5	65%	0.654
Random Forests(10 features)	60%	0.589	55%	0.555

Table VI. Accuracies from cropped (56x56) tumor patches using vgg-f network

Classifier used	***Symmetric uncertainty feature selector***	***AUC***	***Relief-f feature selector***	***AUC***
Naïve bayes (5 features)	47.5%	0.475	55%	0.638
Naïve bayes (10 features)	60%	0.589	52.5%	0.513
Nearest neighbor(5 features)	62.5%	0.666	52.5%	0.523
Nearest neighbor(10 features)	72.5%	0.725	57.5%	0.566
Decision tree(5 features)	70%	0.724	67.5%	0.675
Decision tree(10 features)	**77.5**%	0.713	40%	0.4
Random Forests(5 features)	72.5%	0.725	67.5%	0.675
Random Forests(10 features)	72.5%	0.645	55%	0.555

Table VII. Accuracies by merging warped (vgg-F) and quantitative features from [16]

Classifier used	***Symmetric uncertainty feature selector***	***AUC***	***Relief-f feature selector***	***AUC***
Naïve bayes	70%	0.724	72.5%	0.645
Nearest neighbor	67.5%	0.675	65%	0.654
Decision tree	75%	0.75	72.5%	0.645
Random Forests	**80**%	0.8	77.5%	0.7

Table VIII. Accuracies by merging cropped (40x40) (vgg-F) and quantitative features from [16]

Classifier used	***Symmetric uncertainty feature selector***	***AUC***	***Relief-f feature selector***	***AUC***
Naïve bayes	60%	0.589	75%	0.75
Nearest neighbor	65%	0.654	67.5%	0.675
Decision tree	72.5%	0.773	72.5%	0.725
Random Forests	**80**%	0.8	75%	0.75

Table IX. Accuracies by merging cropped (56x56) (vgg-F) and quantitative features from [16]

Classifier used	***Symmetric uncertainty feature selector***	***AUC***	***Relief-f feature selector***	***AUC***
Naïve bayes	62.5%	0.666	67.5%	0.675
Nearest neighbor	**82.5**%	0.778	65%	0.617
Decision tree	80%	0.651	75%	0.75
Random Forests	80%	0.8	75%	0.75

Table X. Accuracies from warped tumor patches using vgg-m pre-trained network

Classifier used	***Symmetric uncertainty feature selector***	***AUC***	***Relief-f feature selector***	***AUC***
Naïve bayes (5 features)	25%	0.188	45%	0.378
Naïve bayes (10 features)	27.5%	0.203	52.5%	0.548
Nearest neighbor(5 features)	30%	0.30	47.5%	0.475
Nearest neighbor(10 features)	35%	0.35	**57.5**%	0.**575**
Decision tree(5 features)	37.5%	0.33	35%	0.35
Decision tree(10 features)	40%	0.353	20%	0.194
Random Forests(5 features)	30%	0.286	45%	0.403
Random Forests(10 features)	32.5%	0.295	52.5%	0.495

Table XI. Accuracies from cropped (40x40) tumor patches using vgg-m pre-trained network

Classifier used	***Symmetric uncertainty feature selector***	***AUC***	***Relief-f feature selector***	***AUC***
Naïve bayes (5 features)	52.5%	0.483	65%	0.675
Naïve bayes (10 features)	55%	0.513	**67.5**%	0.60
Nearest neighbor(5 features)	47.5%	0.475	62.5%	0.625
Nearest neighbor(10 features)	47.5%	0.475	65%	0.65
Decision tree(5 features)	40%	0.323	57.5%	0.54.
Decision tree(10 features)	37.5%	0.299	52.5%	0.553
Random Forests(5 features)	50%	0.444	62.5%	0.704
Random Forests(10 features)	47.5%	0.436	62.5%	0.675

Table XII. Accuracies from cropped (56x56) tumor patches using vgg-m pre-trained network

Classifier used	***Symmetric uncertainty feature selector***	***AUC***	***Relief-f feature selector***	***AUC***
Naïve bayes (5 features)	45%	0.393	57.5%	0.543
Naïve bayes (10 features)	50%	0.463	52.5%	0.495
Nearest neighbor(5 features)	47.5%	0.475	47.5%	0.475
Nearest neighbor(10 features)	55%	0.55	50%	0.50
Decision tree(5 features)	40%	0.369	**60**%	0.455
Decision tree(10 features)	50%	0.468	55%	0.386
Random Forests(5 features)	45%	0.394	52.5%	0.505
Random Forests(10 features)	45%	0.516	52.5%	0.477

Table XIII. Accuracies by merging warped (vgg-m) and quantitative features from [16]

Classifier used	***Symmetric uncertainty feature selector***	***AUC***	***Relief-f feature selector***	***AUC***
Naïve bayes	42.5%	0.253	65%	0.698
Nearest neighbor	55%	0.55	60%	0.60
Decision tree	60%	0.569	45%	0.49
Random Forests	**62.5**%	0.686	60%	0.676

Table XIV. Accuracies by merging cropped(40x40) (vgg-m) and quantitative features from [16]

Classifier used	***Symmetric uncertainty feature selector***	***AUC***	***Relief-f feature selector***	***AUC***
Naïve bayes	50%	0.403	60%	0.53
Nearest neighbor	40%	0.40	50%	0.50
Decision tree	42.5%	0.341	**77.5**%	0.**713**
Random Forests	62.5%	0.605	62.5%	0.579

Table XV. Accuracies by merging cropped (56x56) (vgg-m) and quantitative features from [16]

Classifier used	***Symmetric uncertainty feature selector***	***AUC***	***Relief-f feature selector***	***AUC***
Naïve bayes	42.5%	0.333	65%	0.635
Nearest neighbor	42.5%	0.425	62.5%	0.625
Decision tree	57.5%	0.40.4	**70%**	0.641
Random Forests	62.5%	0.609	57.5%	0.599

Table XVI. Accuracies from warped tumor patches using vgg-s pre-trained network

Classifier used	***Symmetric uncertainty feature selector***	***AUC***	***Relief-f feature selector***	***AUC***
Naïve bayes (5 features)	40%	0.323	42.5%	0.425
Naïve bayes (10 features)	42.5%	0.341	45%	0.49
Nearest neighbor(5 features)	37.5%	0.299	45%	0.394
Nearest neighbor(10 features)	35%	0.35	**50**%	**0.50**
Decision tree(5 features)	20%	0.188	40%	0.369
Decision tree(10 features)	25%	0.194	32.5%	0.302
Random Forests(5 features)	32.5%	0.295	35%	0.35
Random Forests(10 features)	37.5%	0.33	47.5%	0.475

Table XVII. Accuracies from cropped (40x40) tumor patches using vgg-s pre-trained network

Classifier used	***Symmetric uncertainty feature selector***	***AUC***	***Relief-f feature selector***	***AUC***
Naïve bayes (5 features)	52.5%	0.483	62.5%	0.579
Naïve bayes (10 features)	55%	0.513	**67.5**%	0.**60**
Nearest neighbor(5 features)	37.5%	0.33	57.5%	0.544
Nearest neighbor(10 features)	47.5%	0.436	60%	0.569
Decision tree(5 features)	47.5%	0.475	37.5%	0.299
Decision tree(10 features)	45%	0.516	37.5%	0.33
Random Forests(5 features)	40%	0.40	55%	0.513
Random Forests(10 features)	52.5%	0.505	55%	0.55

Table XVIII. Accuracies from cropped (56x56) tumor patches using vgg-s pre-trained network

Classifier used	***Symmetric uncertainty feature selector***	***AUC***	***Relief-f feature selector***	***AUC***
Naïve bayes (5 features)	60%	0.569	72.5%	0.713
Naïve bayes (10 features)	42.5%	0.341	60%	0.563
Nearest neighbor(5 features)	52.5%	0.483	45%	0.394
Nearest neighbor(10 features)	55%	0.513	40%	0.353
Decision tree(5 features)	60%	0.60	**75**%	0.75
Decision tree(10 features)	40%	0.40	60%	0.676
Random Forests(5 features)	52.5%	0.505	65%	0.635
Random Forests(10 features)	50%	0.50	55%	0.55

Table XIX. Accuracies by merging warped (vgg-s) and quantitative features from [16]

Classifier used	***Symmetric uncertainty feature selector***	***AUC***	***Relief-f feature selector***	***AUC***
Naïve bayes	50%	0.69	75%	0.763
Nearest neighbor	62.5%	0.625	70%	0.70
Decision tree	60%	0.684	52.5%	0.525
Random Forests	**80**%	0.875	72.5%	0.783

Table XX. Accuracies by merging cropped (40x40) (vgg-s) and quantitative features from [16]

Classifier used	***Symmetric uncertainty feature selector***	***AUC***	***Relief-f feature selector***	***AUC***
Naïve bayes	75%	0.778	**82.5**%	0.**83**
Nearest neighbor	50%	0.50	70%	0.70
Decision tree	65%	0.698	57.5%	0.575
Random Forests	67.5%	0.744	72.5%	0.725

Table XXI. Accuracies by merging cropped (56x56) (vgg-s) and quantitative features from [16]

Classifier used	***Symmetric uncertainty feature selector***	***AUC***	***Relief-f feature selector***	***AUC***
Naïve bayes	70%	0.79	**77.5**%	0.795
Nearest neighbor	55%	0.55	72.5%	0.725
Decision tree	65%	0.65	67.5%	0.709
Random Forests	**70**%	0.741	**77.5**%	0.**821**

B. Results obtained from post-relu features

Table XXII. Accuracies from warped tumor patches using vgg-f pre-trained network

Classifier used	***Symmetric uncertainty feature selector***	***AUC***	***Relief-f feature selector***	***AUC***
Naïve bayes (5 features)	52.5%	0.698	50%	0.628
Naïve bayes (10 features)	62.5%	0.694	57.5%	0.673
Nearest neighbor(5 features)	55%	0.55	35%	0.35
Nearest neighbor(10 features)	57.5%	0.575	45%	0.45
Decision tree(5 features)	**65**%	0.625	47.5%	0.563
Decision tree(10 features)	52.5%	0.53	50%	0.509
Random Forests(5 features)	52.5%	0.596	52.5%	0.466
Random Forests(10 features)	40%	0.499	47.5%	0.485

Table XXIII. Accuracies from cropped (40x40) tumor patches using vgg-f pre-trained network

Classifier used	***Symmetric uncertainty feature selector***	***AUC***	***Relief-f feature selector***	***AUC***
Naïve bayes (5 features)	35%	0.35	52.5%	0.466
Naïve bayes (10 features)	42.5%	0.473	37.5%	0.402
Nearest neighbor(5 features)	50%	0.5	42.5%	0.425
Nearest neighbor(10 features)	57.5%	0.575	35%	0.35
Decision tree(5 features)	**62.5**%	0.497	42.5%	0.34
Decision tree(10 features)	52.5%	0.407	30%	0.306
Random Forests(5 features)	45%	0.459	37.5%	0.211
Random Forests(10 features)	45%	0.473	25%	0.211

Table XXIV. Accuracies from cropped (56x56) tumor patches using vgg-f pre-trained network

Classifier used	***Symmetric uncertainty feature selector***	***AUC***	***Relief-f feature selector***	***AUC***
Naïve bayes (5 features)	47.5%	0.415	42.5%	0.405
Naïve bayes (10 features)	57.5%	0.575	50%	0.545
Nearest neighbor(5 features)	60%	0.6	**62.5**%	**0.65**
Nearest neighbor(10 features)	62.5%	0.625	57.5%	0.575
Decision tree(5 features)	52.5%	0.589	35%	0.35
Decision tree(10 features)	47.5%	0.	45%	0.473
Random Forests(5 features)	47.5%	0.477	47.5%	0.454
Random Forests(10 features)	50%	0.5	47.5%	0.459

Table XXV. Accuracies by merging warped (vgg-f) and quantitative features from [16]

Classifier used	***Symmetric uncertainty feature selector***	***AUC***	***Relief-f feature selector***	***AUC***
Naïve bayes	75%	0.875	**90%**	**0.935**
Nearest neighbor	50%	0.495	82.5%	0.825
Decision tree	65%	0.649	67.5%	0.784
Random Forests	65%	0.649	77.5%	0.883

Table XXVI. Accuracies by merging cropped (40x40) (vgg-f) and quantitative features from [16]

Classifier used	***Symmetric uncertainty feature selector***	***AUC***	***Relief-f feature selector***	***AUC***
Naïve bayes	70%	0.81	72.5%	0.74
Nearest neighbor	75%	0.75	65%	0.65
Decision tree	77.5%	0.844	67.5%	0.553
Random Forests	**85%**	0.929	65%	0.731

Table XXVII. Accuracies by merging cropped (56x56) (vgg-f) and quantitative features from [16]

Classifier used	***Symmetric uncertainty feature selector***	***AUC***	***Relief-f feature selector***	***AUC***
Naïve bayes	72.5%	0.868	70%	0.77
Nearest neighbor	70%	0.7	67.5%	0.675
Decision tree	70%	0.763	50%	0.49
Random Forests	**77.5%**	0.82	72.5%	0.773

Table XXVIII. Accuracies from warped tumor patches using vgg-m pre-trained network

Classifier used	***Symmetric uncertainty feature selector***	***AUC***	***Relief-f feature selector***	***AUC***
Naïve bayes (5 features)	42.5%	0.353	65%	0.617
Naïve bayes (10 features)	57.5%	0.575	65%	0.654
Nearest neighbor(5 features)	40%	0.4	52.5%	0.525
Nearest neighbor(10 features)	67.5%	0.675	50%	0.5
Decision tree(5 features)	47.5%	0.446	60%	0.575
Decision tree(10 features)	62.5%	0.653	52.5%	0.539
Random Forests(5 features)	42.5%	0.441	60%	0.589
Random Forests(10 features)	**70**%	0.596	62.5%	0.666

Table XXIX. Accuracies from cropped (40x40) tumor patches using vgg-m pre-trained network

Classifier used	***Symmetric uncertainty feature selector***	***AUC***	***Relief-f fe30ature selector***	***AUC***
Naïve bayes (5 features)	57.5%	0.524	50%	0.525
Naïve bayes (10 features)	57.5%	0.634	37.5%	0.425
Nearest neighbor(5 features)	57.5%	0.575	40%	0.4
Nearest neighbor(10 features)	47.5%	0.475	45%	0.45
Decision tree(5 features)	**82.5**%	0.778	72.5%	0.645
Decision tree(10 features)	62.5%	0.525	67.5%	0.645
Random Forests(5 features)	72.5%	0.804	70%	0.724
Random Forests(10 features)	67.5%	0.7	62.5%	0.62

Table XXX. Accuracies from cropped (56x56) tumor patches using vgg-m pre-trained network

Classifier used	***Symmetric uncertainty feature selector***	***AUC***	***Relief-f feature selector***	***AUC***
Naïve bayes (5 features)	52.5%	0.444	40%	0.381
Naïve bayes (10 features)	55%	0.604	52.5%	0.438
Nearest neighbor(5 features)	55%	0.555	42.5%	0.425
Nearest neighbor(10 features)	50%	0.5	45%	0.45
Decision tree(5 features)	**65**%	0.605	35%	0.283
Decision tree(10 features)	47.5%	0.505	45%	0.477
Random Forests(5 features)	57.5%	0.566	40%	0.4
Random Forests(10 features)	45%	0.429	42.5%	0.453

Table XXXI. Accuracies by merging warped (vgg-m) and quantitative features from [16]

Classifier used	***Symmetric uncertainty feature selector***	***AUC***	***Relief-f feature selector***	***AUC***
Naïve bayes	65%	0.793	70%	0.681
Nearest neighbor	**87.5**%	0.875	65%	0.65
Decision tree	77.5%	0.803	62.5%	0.581
Random Forests	80%	0.885	65%	0.764

Table XXXII. Accuracies by merging cropped (40x40) (vgg-m) and quantitative features from [16]

Classifier used	***Symmetric uncertainty feature selector***	***AUC***	***Relief-f feature selector***	***AUC***
Naïve bayes	70%	0.745	72.5%	0.798
Nearest neighbor	72.5%	0.725	65%	0.65
Decision tree	**80**%	0.651	77.5%	0.789
Random Forests	**80**%	0.885	75%	0.805

Table XXXIII. Accuracies by merging cropped (56x56) (vgg-m) and quantitative features from [16]

Classifier used	***Symmetric uncertainty feature selector***	***AUC***	***Relief-f feature selector***	***AUC***
Naïve bayes	**77.5**%	0.85	72.5%	0.735
Nearest neighbor	67.5%	0.675	67.5%	0.675
Decision tree	72.5%	0.778	55%	0.638
Random Forests	**77.5**%	0.798	65%	0.735

Table XXXIV. Accuracies from warped tumor patches using vgg-s pre-trained network

Classifier used	***Symmetric uncertainty feature selector***	***AUC***	***Relief-f feature selector***	***AUC***
Naïve bayes (5 features)	60%	0.59	55%	0.563
Naïve bayes (10 features)	52.5%	0.593	67.5%	0.75
Nearest neighbor(5 features)	47.5%	0.475	57.5%	0.575
Nearest neighbor(10 features)	52.5%	0.525	60%	0.6
Decision tree(5 features)	37.5%	0.561	45%	0.411
Decision tree(10 features)	47.5%	0.394	60%	0.608
Random Forests(5 features)	60%	0.631	57.5%	0.55
Random Forests(10 features)	50%	0.555	**70**%	0.704

Table XXXV. Accuracies from cropped (40x40) tumor patches using vgg-s pre-trained network

Classifier used	***Symmetric uncertainty feature selector***	***AUC***	***Relief-f feature selector***	***AUC***
Naïve bayes (5 features)	30%	0.265	40%	0.388
Naïve bayes (10 features)	37.5%	0.383	32.5%	0.285
Nearest neighbor(5 features)	42.5%	0.425	40%	0.4
Nearest neighbor(10 features)	**60**%	0.6	40%	0.4
Decision tree(5 features)	42.5%	0.295	52.5%	0.477
Decision tree(10 features)	30%	0.26	47.5%	0.464
Random Forests(5 features)	25%	0.231	35%	0.434
Random Forests(10 features)	32.5%	0.314	37.5%	0.351

Table XXXVI. Accuracies from cropped (56x56) tumor patches using vgg-s pre-trained network

Classifier used	***Symmetric uncertainty feature selector***	***AUC***	***Relief-f feature selector***	***AUC***
Naïve bayes (5 features)	50%	0.51	52.5%	0.513
Naïve bayes (10 features)	55%	0.515	47.5%	0.468
Nearest neighbor(5 features)	50%	0.5	52.5%	0.525
Nearest neighbor(10 features)	55%	0.55	52.5%	0.525
Decision tree(5 features)	60%	0.505	**65**%	0.57
Decision tree(10 features)	62.5%	0.553	62.5%	0.619
Random Forests(5 features)	52.5%	0.489	35%	0.495
Random Forests(10 features)	52.5%	0.523	45%	0.459

Table XXXVII. Accuracies by merging warped (vgg-s) and quantitative features from [16]

Classifier used	***Symmetric uncertainty feature selector***	***AUC***	***Relief-f feature selector***	***AUC***
Naïve bayes	70%	0.733	75%	0.835
Nearest neighbor	72.5%	0.725	75%	0.75
Decision tree	62.5%	0.583	60%	0.523
Random Forests	**77.5**%	0.877	67.5%	0.87

Table XXXVIII. Accuracies by merging cropped (40x40) (vgg-s) and quantitative features from [16]

Classifier used	***Symmetric uncertainty feature selector***	***AUC***	***Relief-f feature selector***	***AUC***
Naïve bayes	62.5%	0.693	67.5%	0.723
Nearest neighbor	65%	0.65	72.5%	0.725
Decision tree	67.5%	0.739	62.5%	0.604
Random Forests	62.5%	0.735	**75**%	0.853

Table XXXIX. Accuracies by merging cropped (56x56) (vgg-s) and quantitative features from [16]

Classifier used	***Symmetric uncertainty feature selector***	***AUC***	***Relief-f feature selector***	***AUC***
Naïve bayes	62.5%	0.725	72.5%	0.83
Nearest neighbor	70%	0.7	62.5%	0.625
Decision tree	60%	0.606	**87.5%**	0.899
Random Forests	67.5%	0.786	80%	0.839

C. Results obtained from multiple slices

TABLE XXXX. Accuracies from warped tumor patches using vgg-f pre-trained network (pre-relu features)

Classifier used	***Symmetric uncertainty feature selector***	***AUC***	***Relief-f fe30ature selector***	***AUC***
Naïve bayes (5 features)	62.5%	0.525	57.5%	0.566
Naïve bayes (10 features)	65%	0.617	50%	0.5
Nearest neighbor(5 features)	62.5%	0.666	52.5%	0.513
Nearest neighbor(10 features)	57.5	0.575	55%	0.638
Decision tree(5 features)	82.5	0.778	72.5%	0.773
Decision tree(10 features)	80	0.651	70%	0.724
Random Forests(5 features)	82.5	0.778	70%	0.724
Random Forests(10 features)	**85**	0.929	72.5%	0.645

TABLE XXXXI. Accuracies from warped tumor patches using vgg-f pre-trained network (post-relu features)

Classifier used	***Symmetric uncertainty feature selector***	***AUC***	***Relief-f fe30ature selector***	***AUC***
Naïve bayes (5 features)	72.5%	0.773	57.5%	0.566
Naïve bayes (10 features)	65%	0.654	55%	0.638
Nearest neighbor(5 features)	70%	0.77	52.5%	0.489
Nearest neighbor(10 features)	60%	0.589	45%	0.459
Decision tree(5 features)	85%	0.929	70%	0.724
Decision tree(10 features)	82.5%	0.825	70%	0.724
Random Forests(5 features)	**87.5%**	0.899	72.5%	0.645
Random Forests(10 features)	85%	0.929	65%	0.617

TABLE XXXXIII. Accuracies by merging warped (vgg-f) –pre relu features and quantitative features from [16]

Classifier used	***Symmetric uncertainty feature selector***	***AUC***	***Relief-f feature selector***	***AUC***
Naïve bayes	70%	0.77	72.5%	0.773
Nearest neighbor	67.5%	0.675	70%	0.724
Decision tree	**82.5%**	0.825	72.5%	0.645
Random Forests	**82.5%**	0.825	75%	0.75

TABLE XXXXIII. Accuracies by merging warped (vgg-f) –post relu features and quantitative features from [16]

Classifier used	***Symmetric uncertainty feature selector***	***AUC***	***Relief-f feature selector***	***AUC***
Naïve bayes	77.5%	0.82	65%	0.617
Nearest neighbor	70%	0.724	57.5%	0.575
Decision tree	87.5%	0.899	70%	0.596
Random Forests	**90%**	0.938	75%	0.75
